# Pneumopathie infiltrante diffuse secondaire à un syndrome des antisynthétases: à propos d´un cas

**DOI:** 10.11604/pamj.2021.39.30.22654

**Published:** 2021-05-11

**Authors:** Nadia Moussa, Rim Khemakhem, Mouna Snoussi, Wafa Fekih, Zouhir Bahloul, Sami Kammoun

**Affiliations:** 1 Service de Pneumologie, Hôpital Universitaire Hédi Chaker, Sfax, Tunisie,; 2 Service de Médecine Interne, Hôpital Universitaire Hédi Chaker, Sfax, Tunisie

**Keywords:** Syndrome des anti-synthétases, pneumopathie infiltrante diffuse, anti-Jo-1, traitement immunosuppresseur, à propos d’un cas, Antisynthetase syndrome, diffuse infiltrating lung disease, anti-Jo1, immunosuppressive therapy, a case report

## Abstract

Les pneumopathies infiltrantes diffuses (PID) constituent une manifestation fréquente des connectivites. Elles peuvent être révélatrices de la maladie ou survenir au cours du suivi. Le syndrome des anti-synthétases (SAS) est une connectivité auto-immune complexe et hétérogène. Des anticorps de type «anti synthétases», en particulier l'anticorps anti-Jo-1 caractérise ce syndrome. Le pronostic du SAS étant conditionné par la survenue d´une PID et de sa sévérité dictant ainsi la prise en charge thérapeutique du SAS. Nous rapportons l´observation d´une patiente âgée de 57 ans se présentant avec un tableau d´une PID aigue fébrile révélant le diagnostic d´un SAS. L´évolution a été favorable sous boli de corticoïdes associés au cyclophosphamide.

## Abstract (en)

Diffuse infiltrate lung diseases (DILDs) are frequent in patients with connective tissue diseases. They can be suggestive of connective tissue diseases or occur during follow-up. Antisynthetase syndrome (ASS) is a complex and heterogeneous connective tissue disease. Antisynthetase antibodies, in particular the anti-Jo1 antibody, are found in patients with this syndrome. The prognosis of ASS is conditioned by the occurrence of DILD and its severity, thus guiding therapeutic management. We here report the case of a 57-year-old female patient presenting with acute febrile DILD revealing ASS. Outcome was favorable under bolus corticosteroids in combination with cyclophosphamide treatment.

## Introduction

Le syndrome des anti-synthétases (SAS) est une forme rare de myosite de chevauchement, elle associe une myosite, une polyarthrite, un phénomène de Raynaud, une atteinte pulmonaire interstitielle et une hyperkératose fissurée des mains [1, 2]. Des anticorps de type «anti synthétases», en particulier l'anticorps anti-Jo1 caractérise ce syndrome. La prévalence des pneumopathies infiltrantes diffuses (PID) est plus élevée avec la positivité des anticorps anti-Jo-1 [3]. Le pronostic du SAS étant conditionné par la survenue d´une PID et de sa sévérité dictant ainsi la prise en charge thérapeutique du SAS [4]. Nous rapportons un tableau de PID rapidement progressive associée à l´anticorps anti-Jo-1 chez une patiente âgée de 57 ans révélateur d´un syndrome des antisynthétases nécessitant des boli de corticoïdes associés au cyclophosphamide avec une évolution favorable.

## Patient et observation

Il s´agit d´une patiente âgée de 57 ans, aux antécédents de fibrillation auriculaire (FA) de découverte récente. Elle a rapportée, l´installation depuis 1 mois d´une dyspnée d´effort d´aggravation progressive associée des gonalgies et des myalgies des membres inférieurs. Devant l´aggravation de sa dyspnée devenant au repos avec l´apparition d´une douleur retro-sternale associée à une toux dans un contexte fébrile, elle a été hospitalisée. A l´examen, elle a été polypnéique à 35 c/min, tachycarde à110b/min avec une saturation en oxygène à (SaO2) à 89%. Des râles crépitants au niveau des bases pulmonaires, avec des œdèmes des membres inférieurs ont été constatés.

**Figure 1:**
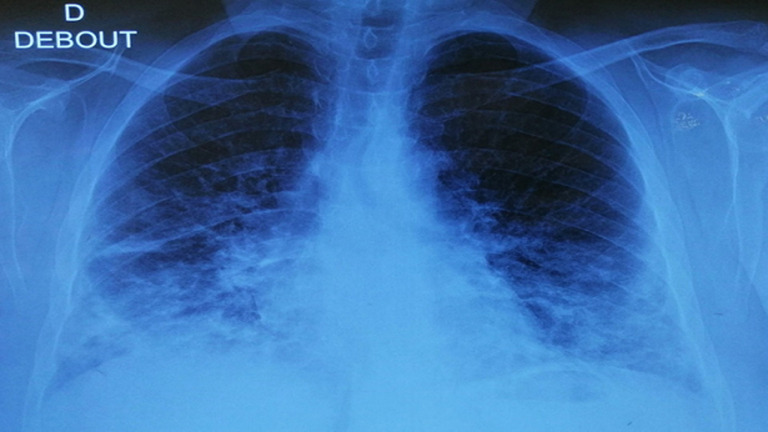
radiographie thoracique de face montrant des opacités alvéolaires et interstitielles prédominant au niveau des bases

**Figure 2:**
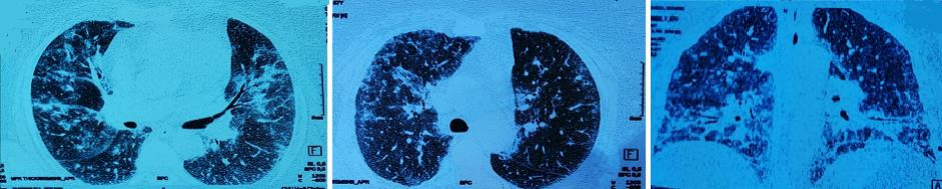
angioscanner thoracique montrant des hypodensités en verre dépoli diffuses aux deux champs pulmonaires plus marquée en inférieurs; condensations parenchymateuses pseudo nodulaires bilatérales, épaississement des lignes septales et des bronchectasies par traction

A la biologie, un syndrome inflammatoire a été noté avec une protéine C-réactive (CRP) à 48 mg/l et une hyperleucocytose à 15200 c/ml. Le taux des éosinophiles, le bilan rénal, hépatique, les taux de créatine-phospho-kinases(CPK), Lactate déshydrogénase (LDH) et les troponines ont été sans anomalies. La radiographie thoracique a montré la présence des opacités pulmonaires et des réticulations prédominent au niveau des bases et sous pleurale étaient présentes à la radiographie thoracique (Figure 1). Un angioscanner thoracique a été réalisé, éliminant une embolie pulmonaire et montrant des hyperdensités en verre dépoli diffuses au deux champs pulmonaires plus marquée au niveau des bases. Des condensations parenchymateuses pseudo nodulaires bilatérales, épaississement des lignes septales et des bronchectasies par traction (Figure 2).

**Figure 3:**
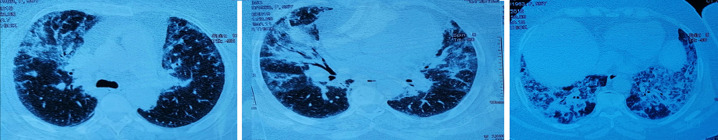
scanner après dégression de la corticothérapie, aggravation radiologique avec un aspect de « crazy paving »

Devant ce tableau de pneumopathie infiltrante diffuse aiguë (PIA), une origine infectieuse était suspectée en premier lieu et elle a été mise sous antibiothérapie à large spectre associée à une oxygénothérapie (12 l/min). Les hémocultures et les sérologies virales et bactériologiques particulièrement pour les atypiques étaient négatifs. La fibroscopie bronchique a été mal tolérée par la patiente. Les anticorps antinucléaires (AAN), les anticorps anticytoplasme des polynucléaires neutrophiles (ANCA), le facteur rhumatoïde et les anti-CCP (antipeptides cycliques citrullinés) étaient négatifs. Les explorations fonctionnelles respiratoires ont conclu à un syndrome restrictif modéré. L´échographie cardiaque trans-thoracique a été sans anomalies. Devant la suspicion de BOOP post-infectieuse et la gravité du tableau clinique, une corticothérapie à la dose de 1 mg/kg/j a été instaurée. L´évolution a été marquée par une amélioration clinique et radiologique. A la dégression de la corticothérapie à une dose de 20mg de prednisolone, elle a présenté une détresse respiratoire nécessitant son hospitalisation de nouveau. Au scanner thoracique, une accentuation des images en verre dépoli et les épaississements des lignes septales réalisant un aspect de « crazy paving » avec des condensations péri-bronchovasculaires et des réticulations intra-lobaires périphériques a été constatée (Figure 3) avec positivité des anticorps anti-antigènes nucléaires solubles de type anti-Jo-1 et Ro52.

**Figure 4:**
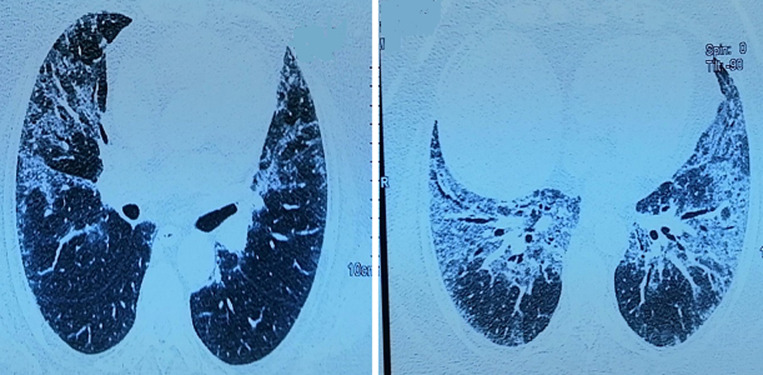
scanner thoracique, régression des condensations après corticothérapie et cyclophosphamide

Une PID rapidement progressive associée à l´anti-Jo1 a été retenue. Elle a reçu des boli de Solumedrol de 1 g associée à un traitement par cyclophosphamide en bolus mensuel de 1 g. L´évolution a été ainsi favorable avec une nette amélioration clinique et une régression modérée en étendue et en densité de la pneumopathie infiltrante diffuse (Figure 4).

## Discussion

Les atteintes interstitielles pulmonaires se présentent principalement sous trois formes: PID aiguë, PID subaiguë/chronique et atteinte interstitielle asymptomatique diagnostiquée sur le scanner thoracique [5]. La PID peut précéder le diagnostic de SAS dans 18% des cas, être de diagnostiqué simultanément que le SAS dans 64% des cas où apparait au cours de l´évolution du SAS dans 18% des cas [5].

Chez notre patiente, l´atteinte interstitielle était révélatrice du diagnostic du SAS. Le diagnostic du SAS a été établi sur l´association d´une PID, arthralgies inflammatoires bilatérales et la positivité de l´anticorps anti-Jo-1. En effet, la présence d´anticorps anti-ARNt synthétases signe le diagnostic devant un tableau clinique et radiologique évocateur. Les plus fréquents de ces auto-anticorps sont les anti-Jo1, les anti-PL7 et les anti-PL12 [6]. Dans notre observation, aucune atteinte musculaire n´a été détectée. Le recours à l´électromyogramme, à l´imagerie musculaire par résonance magnétique ou à l´histologie n´est pas systématique [6]. La fibroscopie bronchique avec lavage bronchiolo-alvéolaire (LBA) est fréquemment réalisée avec des résultats aspécifiques, montrant une alvéolite lymphocytaire habituellement à CD8 ou une alvéolite neutrophilique parfois associée à une éosinophilie [7]. L´histologie n´est pas obligatoire pour porter le diagnostic [4]. Sur le plan scannographique, les deux lésions élémentaires principalement observées dans les PID associées au SAS sont le verre dépoli dans 80% des cas et les réticulations 74% des cas. Une nette prédominance des lésions dans les régions basales et sous-pleurales est constatée [5]. Ainsi, les aspects radiologiques les plus associés au SAS sont la pneumopathie interstitielle non spécifique (PINS) chez 59% des patients, la pneumopathie interstitielle commune chez 23% des patients et la pneumopathie organisée chez 17 % des patients [5]. La pneumopathie organisée était l´aspect radiologique constaté chez notre patiente. En effet, l´atteinte pulmonaire est un facteur pronostic majeur et incite à traiter de manière intensive les patients par corticothérapie systémique, voire d´emblée par association corticoïdes et immunosuppresseurs. La corticothérapie systémique constitue la pierre angulaire du traitement, initialement efficace mais qui nécessite par la suite un complément par immunosuppresseurs dans au moins deux tiers des cas [8]. Les immunosuppresseurs les plus souvent prescrits sont le cyclophosphamide, l´azathioprine, le mycophénolatemofétil, la ciclosporine ou le Tacrolimus [9]. Aucune étude n´a démontré la supériorité de l´un d´entre eux dans le contexte de SAS [4]. Des perfusions d´immunoglobulines sont indiquées en cas de troubles de la déglutition et/ou de pneumopathie d´inhalation [10]. En revanche, dans un nombre limité de cas réfractaires, des résultats prometteurs ont été obtenus avec les anticorps anti-CD20 (Rituximab). Dans une étude ayant inclus 458 patients traités par Rituximab rapporte un taux de réponse global de 78% [11]. Néanmoins, il n´existe aucune comparaison avec les autres traitements immunosuppresseurs disponibles. Chez notre patiente, une amélioration clinique et radiologique a été constatée sous cyclophosphamide. La mise à jour des vaccinations, les vaccinations anti-grippales, anti-Haemophilus et antipneumocoque sont fortement recommandées si les traitements immunosuppresseurs le permettent. La réhabilitation pulmonaire, au même titre que la kinésithérapie musculaire sont bénéfiques.

## Conclusion

Le diagnostic d´une PID dans le cadre d´un SAS doit être suggéré devant un tableau radio-clinique évocateur et sur la présence d´anticorps anti-ARNt synthétases. Selon notre observation, la survenue d´une PID associée à l´anti-Jo-1 incite à être agressif en termes de traitement, avec une association de bolus de corticoïdes et d´immunosuppresseurs.

## References

[1] Hallowell RW, Danoff SK (2014). Interstitial lung disease associated with the idiopathic inflammatory myopathies and the antisynthetase syndrome: recent advances. Current opinion in rheumatology.

[2] Solomon J, Swigris JJ, Brown KK (2011). Myositis-related interstitial lung disease and antisynthetase syndrome. Jornal brasileiro de pneumologia: publicacao oficial da Sociedade Brasileira de Pneumologia e Tisilogia.

[3] Zamora AC, Hoskote SS, Abascal-Bolado B, White D, Cox CW, Ryu JH (2016). Clinical features and outcomes of interstitial lung disease in anti-Jo-1 positive antisynthetase syndrome. Respiratory medicine.

[4] Jouneau S, Hervier B, Jutant EM, Decaux O, Kambouchner M, Humbert M (2015). [Pulmonary manifestations of antisynthetase syndrome]. Revue des maladies respiratoires.

[5] Marie I, Josse S, Hatron PY, Dominique S, Hachulla E, Janvresse A (2013). Interstitial lung disease in anti-Jo-1 patients with antisynthetase syndrome. Arthritis care & research.

[6] Marie I, Josse S, Decaux O, Dominique S, Diot E, Landron C (2012). Comparison of long-term outcome between anti-Jo-1 and anti-PL7/PL12 positive patients with antisynthetase syndrome. Autoimmunity reviews.

[7] Tillie-Leblond I, Wislez M, Valeyre D, Crestani B, Rabbat A, Israel-Biet D (2008). Interstitial lung disease and anti-Jo-1 antibodies: difference between acute and gradual onset. Thorax.

[8] Choy EH, Isenberg DA (2002). Treatment of dermatomyositis and polymyositis. Rheumatology.

[9] Cavagna L, Caporali R, Abdi-Ali L, Dore R, Meloni F, Montecucco C (2013). Cyclosporine in anti-Jo1-positive patients with corticosteroid-refractory interstitial lung disease. The Journal of rheumatology.

[10] Marie I, Hatron PY, Cherin P, Hachulla E, Diot E, Vittecoq O (2013). Functional outcome and prognostic factors in anti-Jo-1 patients with antisynthetase syndrome. Arthritis research & therapy.

[11] Fasano S GP, Hajji R, Loyo E, Isenberg DA (2017). Rituximab in the treatment of inflammatory myopathies: a review. Rheumatology.

